# Effectiveness of the Mindful Motherhood Support Program on Quality of Life Among Palestinian Mothers of Children with Autism Spectrum Disorder: A Quasi-Experimental Study

**DOI:** 10.3390/ijerph23010014

**Published:** 2025-12-20

**Authors:** Bashaer Al-Natsheh, Asma Imam

**Affiliations:** Faculty of Public Health, AL-Quds University, Jerusalem 51000, Palestine; aimam@staff.alquds.edu

**Keywords:** quality of life, mothers, caregiving burden, children, autism spectrum disorder, intervention, support program, effectiveness, Palestine

## Abstract

Raising a child with autism spectrum disorder (ASD) presents severe difficulties that adversely affect the mother’s quality of life (QoL). However, very limited research has examined the impacts of support programs on the QoL of mothers of children with ASD in the Palestinian context. This study assesses the effectiveness of Mindful Motherhood, a comprehensive intervention, in improving QoL in this population. A quasi-experimental design was employed, with 56 mothers assigned to experimental or control groups. Quality of Life (QoL) was measured using the WHOQOL-BREF at baseline, post-intervention, and at a six-month follow-up. The 12-week group-based program led to significant improvements in all QoL domains for the experimental group compared to the controls, with the highest effect sizes in psychological (|δ| = 0.70) and overall QoL (|δ| = 0.68) domains; furthermore, these gains were largely sustained at the six-month follow-up assessment. The results are expected to inform policymakers in developing sustainable support systems for mothers and promoting inclusive, family-centered approaches to ASD care in the Palestinian context.

## 1. Introduction

Autism spectrum disorder (ASD) is a pervasive neurodevelopmental condition characterized by persistent difficulties in social interaction and communication, as well as restricted and repetitive behaviors or interests [1]. Parents experience several difficulties upon learning that their children have ASD [2,3,4], with research indicating that they tend to feel overwhelmed as they must dedicate significant time and energy to caring for their child and teaching essential life skills, which can lead to physical and psychological health problems [5,6,7]. Research has shown that parents of children with ASD commonly experience high levels of stress, anxiety, depression, frustration, and guilt [8,9,10]. These challenges are particularly pronounced among mothers, who frequently bear the primary caregiving role and face unique psychological and social burdens, especially in the Palestinian context, where mothers face ongoing challenges in meeting their child’s medical, developmental, and educational needs while also coping with caregiving responsibilities, financial hardship, and social stigma. Additional barriers include delayed diagnosis, a shortage of qualified professionals, and limited access to evidence-based services, all of which contribute to confusion and dissatisfaction [11,12,13,14]. Collectively, these pressures can severely impact mothers’ overall quality of life (QoL) [15,16]. The World Health Organization (WHO) defines QoL as a multidimensional construct reflecting an individual’s perception of their life within their cultural and value systems, encompassing physical health, psychological state, social relationships, and environmental factors [17]. For mothers of children with ASD, these domains are often disrupted, necessitating targeted interventions to restore balance [18,19]. Empirical studies have underscored that continuous support addressing these areas through psychoeducation, peer networks, and stress management training can significantly enhance maternal well-being and, consequently, family stability [20,21,22]. Research has shown that psychoeducational programs can significantly reduce stress by equipping caregivers with the knowledge and skills needed to cope with ASD-related problems [20,23]. It has been observed that parents can overcome problems through problem-focused coping methods, which positively affects their quality of life [24,25,26,27].

While a robust body of literature has examined support for mothers of children with ASD in an international context [21,28], research focusing specifically on Arab countries is notably limited [23]. A systematic scoping review by Alallawi et al. (2020) initially highlighted significant gaps and the weak quality of existing studies in Arab countries, calling for more high-quality, culturally sensitive research [23,29]. In the Palestinian context, the needs of mothers raising children with ASD remain critically under-addressed, with minimal formal or community-based support available to alleviate their caregiving while the analytical study by Assaf et al. investigates into the family culture surrounding children with ASD in Palestine, underscoring the critical role of familial support systems and the complex cultural dynamics these mothers navigate [30]. Furthermore, emerging graduate research has directly explored the quality of life and coping mechanisms of Palestinian mothers of children with ASD, consistently documenting high levels of stress and a pronounced need for formal support [31,32]. Our study contributes directly to this gap by developing, implementing, and rigorously evaluating the effectiveness of the Mindful Motherhood program, in terms of the improvement of overall QoL in mothers raising children with ASD. This study further examines the program’s impacts on the physical, psychological, social, and environmental domains of QoL, including an assessment of the consistency of these effects at six months post-intervention. It is hypothesized that participants in the intervention group will exhibit statistically significant improvements across all QoL domains compared to a non-intervention control group, with these gains maintained at the six-month follow-up assessment. The findings are expected to inform policymakers in developing sustainable support systems that empower mothers promote more inclusive, family-centered approaches to ASD care within the Palestinian context.

## 2. Materials and Methods

### 2.1. Study Design

This study utilized a quasi-experimental design to assess the impact of the support program on the quality of life of mothers of children with ASD. This approach was selected for its robustness and practicality in evaluating interventions within real-world, resource-limited community settings without randomization where random assignment is often not feasible or ethical [33]. Quasi-experimental designs are recognized as a key methodology for generating evidence of intervention effectiveness when randomized controlled trials are not possible, as they allow for the estimation of causal effects through the use of pre-existing groups and controlled comparisons [34,35].

Participants were split into control and experimental groups. QoL was measured for both intervention and control groups at three times: before the program’s implementation, at two weeks after completing the intervention and a follow-up assessment conducted six months later to evaluate long-term effects. Meanwhile, the control group received no support program, after which a post-test assessment was performed.

This study was conducted at two purposively selected locations to ensure diverse representation of Palestinian communities: Hope Flowers School in Bethlehem (recruiting mothers from the southern West Bank) and Jannati Academy Center (recruiting mothers from the central and northern West Bank). The study followed a longitudinal design. A baseline assessment was conducted on 25 May 2024, prior to the intervention. The program was then implemented from 25 May to 15 September 2024. Follow-up questionnaires were administered at two weeks (1 October 2024) and six months (March 2025) post-intervention.

### 2.2. Study Sample

Purposive sampling was used to select participants, mostly with the assistance of healthcare professionals at different rehabilitation centers that serve children with ASD, who notified prospective participants of the study. Mothers were eligible to participate if they had at least one child aged 4–12 years with a confirmed clinical diagnosis of ASD and provided informed consent. A total of 56 mothers consented to participate in this study, recruited from the South (*n* = 30) and the combined Middle and Northern regions (*n* = 26). Participants from each region were then evenly divided into experimental and control groups, resulting in 15 per group from the South and 13 per group from the Middle/North.

The sample size was calculated using G*Power version 3.1.9.2 [36,37]. The minimum sample size for each of the two groups was calculated as 26, based on the two-tailed test of the difference between two independent means with a 1:1 ratio, test power of 0.80, significance level of 0.05, and a hypothesized large effect size (Cohen’s d = 0.80), based on the comprehensive nature of the intervention. Thus, the analysis indicated a required total sample size of 52. To account for the potential lower efficiency of the non-parametric Mann–Whitney U test, this number was inflated by approximately 15%, yielding a target sample size of 60. Although we recruited 60 participants, 2 participants from each group withdrew from the study, resulting in a final analyzable sample of 56. This final sample was deemed sufficient, as it closely approximated the initial target.

### 2.3. Instruments

#### 2.3.1. Demographic Questionnaire

A structured demographic questionnaire was developed to collect socio-demographic and health-related information from mothers and their children diagnosed with ASD. Maternal variables included age, education level, marital status, employment status, family income, and place of residence. Household composition was also recorded; specifically the number of adults over 18 years, the number of children under 18, and the number of children diagnosed with ASD.

Child-related variables encompassed gender, current age, age at diagnosis, the duration between symptom recognition and formal diagnosis, and health coverage status. These data were collected at the pretest stage and provided a comprehensive profile of participants, allowing for examination of potential associations with quality-of-life outcomes.

#### 2.3.2. World Health Organization Quality of Life Assessment–Brief (WHOQOL-BREF)

The World Health Organization Quality of Life Assessment–Brief (WHOQOL-BREF) was used to evaluate mothers’ perceived quality of life [38]. This instrument consists of 26 items across four domains: Physical health (7 items), psychological well-being (6 items), social relationships (3 items), and environmental health (8 items).

Validity and reliability were assessed for the questionnaire. It was piloted with 10 mothers for clarity, and its content and face validity were confirmed through expert review. Internal consistency was confirmed using Cronbach’s alpha, which indicated a high overall reliability of 0.94, while domain-specific Cronbach’s alpha coefficients were 0.85 for physical, 0.84 for psychological, 0.72 for social, and 0.86 for environmental health. The test–retest reliability was evaluated by re-administering the questionnaire to the same group 15 days later, showing significant correlation across all items.

### 2.4. Ethical Considerations

Approval to conduct the study was obtained from the Research Ethical Review Committee at Al-Quds University, Palestine. Mothers received a clear explanation of the study’s nature and objectives and were informed that participation is voluntary, with the right to withdraw without consequence, and that all collected data would be treated confidentially. Informed consent was obtained from the participating mothers after they agreed to take part in the study. Following data collection, de-identified data were entered into SPSS and stored in secure files on the researcher’s computer with restricted access. The researcher stored hard copies of the completed data sheets in a locked file cabinet. The research team only used the data for research purposes.

### 2.5. Developing the Intervention—Mindful Motherhood: A Support and Education Program for Mothers Raising Children with ASD

The Mindful Motherhood program is a comprehensive 12–14-week support and education initiative designed specifically for mothers of children with ASD. Developed through collaboration with psychological counselors and experts in family therapy, the program integrates relevant research outcomes to address the emotional, cognitive, and social challenges faced by these mothers [21,22,23,25,34,39]. Drawing from QoL research and the psychological counseling literature, the program provides a structured yet flexible framework to enhance maternal well-being, foster resilience, and build a supportive community.

Each weekly session lasts two hours and is facilitated by two trained professionals, ensuring a balanced approach between psychoeducation, group discussion, and skill-building exercises. The program maintains a small group setting of 13–15 participants to promote trust, confidentiality, and meaningful engagement. Attendance is carefully monitored to preserve group cohesion, as consistent participation is key to the program’s effectiveness.

Crucially, the program was culturally and contextually adapted for Palestinian mothers. This included integrating discussions on navigating social stigma and leveraging extended family networks, framing coping strategies within cultural and religious values, and focusing on practical problem-solving tailored to a context with limited access to professional resources and financial hardship. In addition, recruitment for the intervention was conducted during a period of political instability in Palestine. Consequently, we enrolled only mothers who could safely and reliably access the program site to ensure consistent participation.

### 2.6. Intervention Description

The Mindful Motherhood program is a structured, group-based support intervention designed to holistically support mothers of children with ASD. The program is sequenced into three progressive phases to mirror the natural caregiving journey. The initial foundation phase (Sessions 1–3) focuses on building a safe and trusting environment by providing education about ASD and validating common experiences of grief and loss, thereby guiding mothers toward acceptance. This foundational work is essential for engaging in the subsequent skill-building phase (Sessions 4–9), which empowers mothers by teaching practical, actionable skills in cognitive restructuring, self-care, boundary setting, communication, and stress management, moving beyond talk therapy to ensure tangible outcomes.

The final phase (Sessions 10–12) focuses on integration and future-oriented growth, helping mothers to cultivate a positive self-concept, manage future concerns, and instill a sense of hope for sustainable well-being. Throughout all phases, the program adopts a multi-dimensional approach, simultaneously addressing cognitive, emotional, behavioral, social, and physical aspects of the mothers’ lives. Furthermore, the group format itself is a key intervention component, actively building a vital peer support network to combat isolation. In essence, the program’s compassionate focus is not on changing the child with autism, but on strengthening the mother as the primary caregiver by equipping her with the emotional tools, practical skills, and social support necessary to navigate her challenges with greater resilience and well-being. Table A1 in Appendix A details all session agendas throughout the program. Ultimately, Mindful Motherhood aims to shift mothers from survival mode to empowered advocacy, fostering resilience that benefits both themselves and their children with ASD.

### 2.7. Statistical Analysis

Statistical analysis was performed using IBM SPSS Statistics, version 23, with a significance level set at *p* = 0.05. Descriptive statistics are used to summarize the data, including frequencies and percentages for categorical variables and means with standard deviations for continuous variables. The Shapiro–Wilk test was conducted to assess the normality of the data, which yielded a significant result (*p* = 0.025), thus indicating that the data were not normally distributed. Accordingly, nonparametric tests were used for subsequent analysis. Within-group comparisons of QoL scores were conducted using Wilcoxon Signed-Rank Tests. Between-group comparisons at three time points (pre-intervention, post-intervention, and six-month follow-up) were performed using the Mann–Whitney U test. Effect sizes for between-group comparisons were calculated using Cliff’s Delta (δ), with the following conventional thresholds applied: |δ| = 0.147 (small), 0.147–0.33 (medium), and ≥0.33 (large). Additionally, the Friedman test followed by the Wilcoxon sign test was applied to assess changes in QoL scores across the three time points within each group.

## 3. Results

### 3.1. Descriptive Analysis

Table 1 presents the sociodemographic characteristics of the study sample, divided by group (Experimental, *n* = 28; Control, *n* = 28). The majority (approximately 59%) were aged 30–39 years. In terms of education, 37.5% held a bachelor’s degree, followed by 30.1% with secondary school education or less. Geographically, 50.0% were from southern governorates, 26.8% from central governorates, and 23.2% from northern governorates. Regarding residence, 53.6% lived in cities, 41.1% in villages, and 5.4% in refugee camps.

Most participants (92.9%) were married, and 69.6% were unemployed. Additionally, the largest subgroup (37.5%) had three children under the age of three. The vast majority (92.9%) had one child diagnosed with autism. Monthly family income was reported as moderate by 58.9%, while 21.4% reported it as below or well below average. Regarding the diagnosis timeline, 60.7% indicated that less than two years passed between noticing developmental concerns and receiving an ASD diagnosis. Additionally, 55.4% stated that their child was diagnosed at age three or older, while 44.6% received the diagnosis before the age of three.

Table 2 presents the median and interquartile range (IQR) for QoL domains at baseline, post-intervention, and at the 6-month follow-up. The data reveal a clear divergent trend between the control and experimental groups. In the control group, median scores across all domains—namely, Physical, Environmental, Psychological, Social, and Overall QoL—demonstrated a slight decline or remained relatively stable from pre-intervention to the 6-month follow-up. In stark contrast, the experimental group showed a marked improvement immediately after the intervention, with substantially higher median scores post-intervention compared to their baseline. Notably, these gains in the experimental group were largely sustained at the 6-month follow-up, with scores remaining well above both their own baseline levels and the corresponding scores of the control group at the same time point. This pattern suggests a positive and persistent effect of the intervention on QoL outcomes.

### 3.2. Between-Group Comparisons of Overall QoL and Its Domains

Table 3 shows the non-parametric test results. Mann–Whitney U tests were conducted to evaluate the differences in QoL domain scores between the experimental and control groups across three time points: pre-intervention, post-intervention, and at the 6-month follow-up. At baseline, there were no significant differences between the groups in any QoL domain (all Cliff’s Delta |δ| < 0.147, negligible effect sizes), indicating that the groups were equivalent before the intervention.

Following the intervention, the experimental group demonstrated statistically significant and large improvements compared to the control group across all four QoL domains and overall QoL at the post-test assessment (all *p* < 0.05; Physical: δ = −0.64; Environmental: δ = −0.66; Psychological: δ = −0.70; Social: δ = −0.47; Overall QoL: δ = −0.68). These substantial gains were largely maintained at the 6-month follow-up, with large effect sizes persisting for the physical (δ = −0.63), environmental (δ = −0.59), psychological (δ = −0.70), and overall QoL (δ = −0.68) domains. Although the social domain showed a sustained improvement, the effect size decreased from large to medium (δ = −0.34).

### 3.3. Within-Group Changes in Quality-of-Life Scores from Baseline to Post-Intervention and 6-Month Follow-Up

To analyze changes over time within each group, Friedman tests were conducted for each QoL domain, followed by Wilcoxon signed-rank post hoc tests as shown in Table 4.

The experimental group showed a significant overall change in physical scores over time (*p* = 0.005), with a highly significant improvement immediately after the intervention (*p* < 0.001). This improvement was maintained at the 6-month follow-up and remained significant in comparison to baseline (*p* = 0.015). No significant change was observed between the end of the intervention and the 6-month mark, indicating a stable improvement. There was no significant change in physical domain in the control group.

In the psychological domain, there was a significant overall change in the experimental group (*p* < 0.001), with the most significant improvement between before and after the intervention (*p* < 0.001). Although the improvement was still statistically significant at six months, there was a trend towards a decline (*p* = 0.043). In contrast, the control group showed no significant change over time in this domain.

Although the overall test for the environmental domain was not significant (*p* = 0.120), the Pre vs. Post difference in the experimental group was significant (*p* = 0.004); meanwhile, the control group showed no significant difference. In addition, a significant improvement in social outcomes was observed in the experimental group (*p* = 0.41), with a significant improvement observed between the pre- and post-intervention timepoints (*p* = 0.017). However, this improvement did not persist at six months. There was no significant difference in the control group between all three time points.

The results demonstrate a significant change in the overall QoL (total score) in the experimental group (*p* < 0.001), with a large and immediate improvement between the pre- and post-intervention timepoints (*p* < 0.001). However, a marginal or borderline significant decline from the post-intervention peak to the 6-month follow-up was observed, indicating that some of the peak benefits were not fully maintained. In contrast, the control group showed no significant change over time, as per the Friedman analysis.

## 4. Discussion

This study examined the effectiveness of the Mindful Motherhood intervention among mothers of children with ASD in Palestine. The post-test QoL scores for the experimental group increased significantly, with the most significant improvement in psychological and overall QoL domains, while those for the control group remained stable or showed slight declines. These results are consistent with the findings of a study conducted in Jordan—which is similar to the Palestinian context—demonstrating the effectiveness of structured support interventions in improving QoL among parents of children with ASD [25]. The significant improvement in QoL could be attributed to the nature of the program, as a comprehensive and holistic intervention that addresses the multifaceted areas of QoL. The findings are further supported by another study reporting that parents of children with ASD need ongoing support for their physical, psychological, social, and environmental health, which can improve families’ quality of life and ability to address behavioral difficulties [40,41].

By acquiring knowledge, engaging in psychoeducation, enhancing their skills, and developing practices, mothers can formulate coping strategies and transition from emotional distress to fostering hope and empowerment within a group setting [42]. This environment promotes teamwork and interaction, enabling them to cultivate a sense of value within a supportive social context, thus enhancing overall satisfaction and well-being in a therapeutic manner.

At the six-month follow-up evaluation, the intervention’s effectiveness remained significant, with most gains maintained—particularly those in the psychological and overall QoL domains. The effect size remained high, indicating improvement in QoL over time. However, there was some decline in the social and environment domains, suggesting the need for further support to achieve sustained improvements.

The most substantial enhancements were observed in the psychological realm. Initial sessions focused on assisting mothers in accepting their child’s diagnosis and processing grief. Research findings have indicated that Acceptance and Commitment Therapy (ACT) enhances psychological flexibility and alleviates stress, with parents adhering to the ACT protocol reporting notable improvements in psychological flexibility, emotional states, personal values in daily life, and a reduction in parental stress and distress regarding their children’s behaviors [43,44]. Subsequent sessions used cognitive restructuring strategies—a crucial element of cognitive-behavioral therapy (CBT) models—which focus on altering negative thought patterns to alleviate emotional distress. These findings align with recent research examining the efficacy of group counseling rooted in psychoeducation and cognitive behavioral therapy (CBT) in alleviating depression among mothers of children with ASD [45,46,47].

The intervention produced a short-term improvement in social outcomes that disappeared by the 6-month follow-up. Group-based interventions improved social domains and reduced feelings of isolation in mothers by creating social support networks facilitated by peers. These networks provide emotional strength, shared problem-solving, and long-term social bonds, enabling mothers to implement strategies in real life contexts [48]. The program also strengthened family relationships through modules on communication, boundary-setting, and culturally sensitive parenting strategies, leading to better balance between caregiving and personal needs.

The study underscores the significance of social support in alleviating caregiver stress and enhancing mother–child relationships through varied participation. This finding aligns with Rayan & Ahmad (2016) [39], who indicated that scores in the health domain of social relationships were significantly improved following a mindfulness-based intervention, resulting from the non-judgmental acceptance of the child’s condition, while Rezq et al. highlighted the importance of social support in improving mothers’ QoL who are raising children with ASD [39,49].

The impact on the environmental domain was tenuous and inconsistent. A transient effect may have occurred, but it lacked robustness and sustainability. Despite the intervention’s emphasis on imparting practical knowledge regarding ASD, problem-solving skills, advocacy for children’s needs, self-care, boundary-setting, and strategies for fostering more manageable and supportive home environments, the enhancement in this area was not substantial. This finding aligns with a previous study indicating that parents of children with ASD remain unsatisfied with various aspects of the environmental domain, including income, housing circumstances, and healthcare services for their children [39].

Furthermore, maintaining hope is very important. Snyder’s concept of hope is a cognitive behavioral structure with three main components: Goals, agency, and pathways. Success generates positive emotions, while failure leads to negative emotions. Hopeless individuals perceive themselves to have less agency and pathways, causing depression and other negative impacts in mothers with autistic children [50,51]. The most commonly suggested helpful method for parents of children with ASD is to learn how to cope and solve problems. Training parents in this way can lead to healthier children, as it helps to better organize their environment, expand their problem-solving skills, and improve the ability to cope with their situation [52,53].

Ultimately, the Mindful Motherhood program appeared to initiate a positive feedback loop in the lives of participants. By first addressing psychological acceptance and distress, then equipping mothers with practical tools for coping, communication, and self-care, the intervention enabled them to reframe their challenges and make meaningful improvements across all QoL domains. This transformation from surviving to thriving underscores the power of well-designed, culturally sensitive, and group-based psychosocial interventions.

Several limitations of this study should be acknowledged. The use of a quasi-experimental design with non-random assignment, while pragmatic, introduced potential for selection bias. Furthermore, the reliance on self-reported measures for the primary outcome may have led to social desirability bias. The use of purposive sampling also limits the generalizability of the findings to all Palestinian mothers of children with ASD. Future research would benefit from a randomized controlled trial design incorporating more objective biomarkers of stress and well-being.

## 5. Conclusions

Initiatives designed to alleviate stress and enhance the welfare of caregivers of children with ASD provide beneficial results for caregivers and, consequently, for the children themselves. Such an intervention must be a comprehensive procedure that benefits and attends to every family member.

The Mindful Motherhood program was found to be highly effective in improving various QoL domains for mothers of children with ASD, addressing physical, psychological, social, and environmental needs through structured sessions and peer support, helping mothers to transition from crisis management to proactive coping. The peer support component fosters long-term resilience and social connections. Future implementations should consider scaling the program through telehealth and training community health workers, ensuring accessibility in low-resource settings. Local health and education authorities could integrate the Mindful Motherhood curriculum into existing community health centers or parent support networks. Training experienced social workers and special education teachers to be “master trainers” could facilitate the studied intervention’s wider dissemination. Additionally, adapting the program to a hybrid or fully telehealth format could overcome geographical barriers and increase accessibility for mothers in remote villages, ensuring a more family-centered approach to ASD care across Palestine.

## Figures and Tables

**Table 1:** Sociodemographic characteristics of the study sample (Experimental, *n* = 28; Control, *n* = 28).

Variable	Category	Experimental (*n* = 28)	Control (*n* = 28)
Mothers’ Age	20–29 years	6 (21.4%)	4 (14.3%)
	30–39 years	15 (53.6%)	18 (64.3%)
	40–49 years	7 (25.0%)	6 (21.4%)
Level of Education	Secondary School or less	11 (39.3%)	7 (25.0%)
	Diploma	9 (32.1%)	4 (14.3%)
	Bachelor	7 (25.0%)	14 (50.0%)
	High education (MSc/PhD)	2 (7.1%)	3 (10.7%)
Place of Residence (Governorate)	South	14 (50.0%)	14 (50.0%)
	Middle	7 (25.0%)	8 (28.6%)
	North	7 (25.0%)	6 (21.4%)
Type of Residence	City	16 (57.1%)	14 (50.0%)
	Village	11 (39.3%)	12 (42.9%)
	Camp	1 (3.6%)	2 (7.1%)
Marital Status	Married	23 (82.1%)	23 (82.1%)
	Divorced	4 (14.3%)	5 (17.9%)
	Widow	1 (3.6%)	0 (0.0%)
Employment Status	No	22 (78.6%)	17 (60.7%)
	Yes, part-time	3 (10.7%)	5 (17.9%)
	Yes, full-time	3 (10.7%)	6 (21.4%)
Number of Children < 18	Less than 3	12 (42.9%)	8 (28.6%)
	3	9 (32.1%)	12 (42.9%)
	More than 3	7 (25.0%)	8 (28.6%)
Family Income	Within average	15 (53.6%)	18 (64.3%)
	Less/much less than average	8 (28.6%)	6 (21.4%)
	Higher than average	2 (7.1%)	7 (25.0%)

**Table 2:** Median and interquartile range for each QoL domain pre- post intervention and six months follow-up for experimental and control groups.

Domain	Pre-Intervention Median (IQR)		Post-Intervention Median (IQR)		6-Month Follow-Up Median (IQR)	
	Control	Experiment	Control	Experiment	Control	Experiment
Physical	3.00 (2.43–3.64)	3.29 (2.46–3.57)	2.71 (2.21–3.57)	3.71(3.32–4.11)	2.21(1.89–3.14)	3.50 (3.14–4.00)
Psychological	2.44 (1.88–3.00)	2.81 (2.41–3.34)	2.25 (1.75–2.88)	3.38(2.91–3.50)	2.13 (1.56–2.75)	3.13 (2.66–3.50)
Environmental	2.58 (2.04–3.29)	3.00 (2.38–3.42)	2.50 (1.88–2.83)	3.67(3.17–4.00)	2.33 (2.00–3.08)	3.50 (2.67–3.96)
Social	3.00 (2.00–3.58)	3.33 (2.67–3.67)	3.00 (2.00–3.63)	3.67(3.08–4.00)	2.83 (2.00–3.92)	3.33 (3.00–4.00)
Overall	2.69 (2.08–3.32)	3.00 (2.55–3.38)	2.60 (2.13–3.06)	3.60(3.13–3.88)	2.38 (1.88–2.98)	3.46 (2.83–3.78)

**Table 3:** Between-group comparisons of overall QoL and its domains: Experimental vs. Control at Baseline (Pre-I), Post-Intervention (Post-I), and 6-Month Follow-up.

Domain	Time Point	Test Statistic (U, *p*)	Effect Size(δ), 95% CI
Physical	Pre-I	370.00, *p* = 0.812	−0.06 [−0.35, 0.23]
	Post-I	141.50, *p* < 0.001	−0.64 [−0.82, −0.40]
	6-Month	145.50, *p* < 0.001	−0.63 [−0.81, −0.38]
Environmental	Pre-I	241.00, *p* = 0.105	−0.39 [−0.65, −0.08]
	Post-I	131.50, *p* < 0.001	−0.66 [−0.83, −0.43]
	6-Month	162.50, *p* = 0.001	−0.59 [−0.78, −0.33]
Psychological	Pre-I	300.50, *p* = 0.265	−0.23 [−0.52, 0.10]
	Post-I	118.50, *p* < 0.001	−0.70 [−0.86, −0.48]
	6-Month	119.50, *p* < 0.001	−0.70 [−0.86, −0.48]
Social	Pre-I	298.00, *p* = 0.280	−0.24 [−0.53, 0.09]
	Post-I	208.00, *p* = 0.011	−0.47 [−0.70, −0.17]
	6-Month	259.50, *p* = 0.048	−0.34 [−0.60, −0.01]
QoL	Pre-I	295.50, *p* = 0.113	−0.25 [−0.54, 0.08]
	Post-I	124.50, *p* < 0.001	−0.68 [−0.85, −0.45]
	6-Month	124.50, *p* < 0.001	−0.68 [−0.85, −0.45]

A negative δ value indicates that the experimental group had higher median scores on the QoL domain than the control group. The magnitude of the effect is interpreted using the absolute value, |δ|.

**Table 4:** Friedman tests followed by Wilcoxon post-hoc analyses.

Domain	Group	Friedman Test (*p*-Value)	Pre vs. Post (*p*-Value)	Pre vs. 6-Month (*p*-Value)	Post vs. 6-Month (*p*-Value)
Physical	Experimental	0.005 *	0.001 *	0.015 *	0.321
	Control	0.672	0.681	0.399	0.535
Psychological	Experimental	0.001 *	0.001 *	0.043 *	0.080
	Control	0.672	0.681	0.399	0.535
Environmental	Experimental	0.12	0.004 *	0.235	0.235
	Control	0.396	0.949	0.347	0.344
Social	Experimental	0.041 *	0.017 *	0.163	0.320
	Control	0.932	0.428	0.82	0.622
Overall QoL	Experimental	0.001 *	0.001 *	0.021 *	0.050
	Control	0.488	0.738	0.171	0.158

* Significant (*p* < 0.05).

## Data Availability

The data that support the findings of this study are available on request from the corresponding author on reasonable request at besho.natsheh@gmail.com.

## Abbreviations

The following abbreviations are used in this manuscript:

ASD	Autism Spectrum Disorder
QoL	Quality of Life

## Appendix A

**Table A1:** Session agendas for the Mindful Motherhood support program.

Session and Title	Agenda
Preparatory session	Welcome, introductions, and icebreakers. Introduction to the program and group objectives. Setting and discussing group goals. Building a mentorship relationship. Assessing needs. Scheduling suitable days and times for sessions. Reviewing the day and gathering feedback from mothers.
Session One Introduction and education about autism	Welcome, introductions, and ice-breaking. Establishing and discussing group rules. Building relationships. Psychoeducation on ASD, including symptoms, diagnosis, follow-up, prevalence, and causes. Review of the day and feedback from the mothers on the session.
Session two Common responses and the process of loss and grief	Psychoeducation and discussion on common reactions to diagnosis. Psychoeducation on grief and feelings of loss. The process and stages of grief. Sharing mothers’ experiences of loss and grief. The importance of maintaining physical and mental health for mothers. Review of the day and feedback from mothers on the session.
Session Three Acceptance	Psychoeducation on acceptance and related issues. Raising parents’ and siblings’ awareness of common experiences with autistic children. The process of learning unconditional love and letting go of expectations. Practices to promote greater acceptance and gratitude. A conversation on matching everyday expectations with reality. Stress management and life contentment. Review of the session and mothers’ input.
Session Four The Cognitive model and ASD: Cognitive restructuring for acceptance and coping	Cognitive restructuring psychoeducation. Teaching methods of cognitive restructuring to promote acceptance. Cognitive restructuring’s function in daily and long-term stress management. Handling the stress that comes with having a child with autism. Review of the session and mothers’ input.
Session Five The impact of ASD on mothers’ self-care Importance of a balanced diet for mothers and the child with ASD	Psychoeducation and discussion on the impact of ASD on mothers’ personal and emotional well-being. Psychoeducation on self-care for mothers. Assessing the current self-care practices of mothers. Identifying commitments related to improving self-care for each mother. Education on nutritional basics, benefits of a balanced diet, food groups, special dietary needs, healthy eating habits, and nutritional challenges in autism, emphasizing the importance of nutrient-rich foods. Review of the day and feedback from the mothers on the session.
Session Six Establishing healthy boundaries for maternal well-being	Psychoeducation on healthy boundaries and setting boundaries. Encouraging mothers to reflect on their current boundaries with family members and discussing the progress they have made. Discussing boundaries as a form of self-care for mothers. Exploring boundaries to provide participants with space to reflect on their relationship with themselves and family members if changes are made to their current boundaries, and discussing the progress they have achieved. Review of the day and feedback from the mothers on the session.
Session Seven Communication and connection Parenting	Incorporating topics that have been discussed and topics the mothers wish to discuss regarding their child with autism. The importance of communication between mothers and their child with autism. Psychoeducation on communication and interaction skills. Diana Baumrind’s parenting style theory, US statistics, culture-related parenting styles, four types, other parenting styles, common behavioral problems, and recommendations for addressing behavioral issues. Review of the day and feedback from the mothers on the session.
Session Eight Coping and support	Education on psychological stress. Psychoeducation on coping methods, mechanisms, and skills. Identifying the coping skills currently used by participants. Discussing positive and negative coping skills. Psychoeducation on the importance of having a strong support system and equipping them with the necessary skills to build one. Review of the day and feedback from the mothers on the session.
Session Nine Problem-solving and relaxation	Education on problem-solving methods and skills. The importance of making decisions effectively, away from stress and emotional pressure. Education on the importance of relaxation in facing psychological and social stress. Training on relaxation techniques. Review of the day and feedback from the mothers on the session.
Session Ten Positive self-concept and outlook on life	Discussing negative thoughts about oneself and life. Positive practices as alternatives to negative thinking. Deepening the concept of positive self-relationships in forming a positive self, family, and social life perspective. Strengthening and rooting self-empowerment to face life’s challenges. Review of the day and feedback from the mothers on the session.
Session Eleven The future and adopting a better outlook on life	Discussing mothers’ current concerns about the future of their child with autism. Psychoeducation on the shared concerns of mothers and their children with autism. Improving the quality of physical, psychological, and social well-being. Focusing on positive emotions and self-concepts about oneself and life. Review.
Session Twelve Instilling hope and closure	Instilling hope for the future. Inviting participants to reflect on their experience in the group. Concluding the counseling relationship. Ensuring the improvement in the quality of life for the mothers. Reviewing and summarizing the sessions, gathering feedback from the mothers on the session, and concluding.
